# Examination of optimized protocols for pCASL: Sensitivity to macrovascular contamination, flow dispersion, and prolonged arterial transit time

**DOI:** 10.1002/mrm.28839

**Published:** 2021-05-19

**Authors:** Logan X. Zhang, Joseph G. Woods, Thomas W. Okell, Michael A. Chappell

**Affiliations:** ^1^ Institute of Biomedical Engineering, Department of Engineering Science University of Oxford Oxford United Kingdom; ^2^ Wellcome Centre for Integrative Neuroimaging, FMRIB, Nuffield Department of Clinical Neuroscience University of Oxford Oxford United Kingdom; ^3^ Department of Radiology University of California San Diego San Diego California USA; ^4^ Mental Health and Clinical Neuroscience, School of Medicine University of Nottingham Nottingham United Kingdom; ^5^ Sir Peter Mansfield Imaging Centre, School of Medicine University of Nottingham Nottingham United Kingdom; ^6^ Nottingham Biomedical Research Centre, Queen’s Medical Centre University of Nottingham Nottingham United Kingdom

**Keywords:** arterial spin labeling, flow dispersion, macrovascular contamination, optimal experimental design, perfusion, prolonged arrival time

## Abstract

**Purpose:**

Previously, multi‐ post‐labeling delays (PLD) pseudo‐continuous arterial spin labeling (pCASL) protocols have been optimized for the estimation accuracy of the cerebral blood flow (CBF) with/without the arterial transit time (ATT) under a standard kinetic model and a normal ATT range. This study aims to examine the estimation errors of these protocols under the effects of macrovascular contamination, flow dispersion, and prolonged arrival times, all of which might differ substantially in elderly or pathological groups.

**Methods:**

Simulated data for four protocols with varying degrees of arterial blood volume (aBV), flow dispersion, and ATTs were fitted with different kinetic models, both with and without explicit correction for macrovascular signal contamination (MVC), to obtain CBF and ATT estimates. Sensitivity to MVC was defined and calculated when aBV > 0.5%. A previously acquired dataset was retrospectively analyzed to compare with simulation.

**Results:**

All protocols showed underestimation of CBF and ATT in the prolonged ATT range. With MVC, the protocol optimized for CBF only (CBFopt) had the lowest sensitivity value to MVC, 33.47% and 60.21% error per 1% aBV in simulation and in vivo, respectively, among multi‐PLD protocols. All multi‐PLD protocols showed a significant decrease in estimation error when an extended kinetic model was used. Increasing flow dispersion at short ATTs caused increasing CBF and ATT overestimation in all protocols.

**Conclusion:**

CBFopt was the least sensitive protocol to prolonged ATT and MVC for CBF estimation while maintaining reasonably good performance in estimating ATT. Explicitly including a macrovascular component in the kinetic model was shown to be a feasible approach in controlling for MVC.

## 1 INTRODUCTION

Arterial spin labeling (ASL) is gaining popularity for its inherently non‐invasive ability to quantify brain perfusion, that is, regional cerebral blood flow (CBF).^1^ ASL image acquisition adopts a label‐control approach. In the label image, arterial‐blood water spins are labeled by inversion,^2^ followed by one or multiple post‐labeling delays (PLD) to allow blood to perfuse the target tissue before image readout. The same procedure is administered in the control image but without inversion of the spins. An ASL image is obtained by subtracting the label image from the control image. Perfusion quantification using a single PLD is the general recommendation of the community for clinical applications,^3^ due to its robustness to arterial transit time (ATT) and relatively simple implementation. However, there is a trade‐off between the recommended long PLD, which ensures a more complete arrival of blood into long ATT regions, and ASL signal loss due to T_1_ relaxation. Therefore, there has been increasing interest in the greater accuracy available from multi‐PLD ASL.^4^ In multi‐PLD ASL, a series of paired label‐control images can be acquired by incrementing the PLD or by use of a Look‐Locker acquisition scheme.^5^ Signals from different time points are then fit with a tracer kinetic model depicting the dynamic concentration of the labeled blood water,^6^ enabling simultaneous quantification of CBF and other parameters, such as ATT.

Recently, a general framework was proposed by Woods et al for optimizing multi‐PLD sampling protocols.^7^ This framework yielded two optimized multi‐PLD protocols: CBF‐ATTopt and CBFopt, which were optimized for combined CBF and ATT estimation and CBF estimation only, respectively. Both optimized protocols achieved better parameter estimation performance than the more commonly used evenly spaced multi‐PLD and single‐PLD protocols.^7^ However, as multi‐PLD ASL tends to use some short PLDs, when labeled blood may not have reached the target tissue but remains in the major arteries, artifacts caused by macrovascular signal contamination (MVC) might arise.^8^ Furthermore, flow dispersion due to blood traversing the vascular branches and cardiac pulsation will compromise the idealized arterial input function (AIF) in the kinetic model used in this framework.^9^ The effects of macrovascular contamination and flow dispersion might be more prominent in the presence of prolonged ATTs seen in cerebrovascular disease^10^ and deep white matter.^11^

In this study, we tested the performance for CBF and ATT estimation of the previously proposed protocols, CBF‐ATTopt and CBFopt, along with a reference multi‐PLD protocol and a single‐PLD protocol, under the effects of MVC and dispersion over a prolonged ATT range (PAR), using both simulated and in vivo data. We examined CBF and ATT estimation errors across a range of arterial blood volumes (aBVs) to investigate the sensitivity to MVC of each protocol, while also comparing the results with an extended kinetic model that explicitly accounts for MVC effects. Furthermore, estimation errors of each protocol across different levels of dispersion, as well as over a PAR, were also examined.

## 2 METHODS

### 2.1 Kinetic modeling

The kinetic model used in this work was based on the general kinetic model proposed by Buxton et al^6^ incorporating a macrovascular compartment^8^ and a Gamma dispersion kernel.^12^ In the general kinetic model, the ASL difference signal of the tissue compartment $\Delta M_{t}\left(t\right)$ can be expressed by:

$$\Delta M_{t}\left(t\right)=2M_{0B}f\{c\left(t\right)*\left[r\left(t\right)\cdot m\left(t\right)\right],$$

where $M_{0B}$ is the equilibrium magnetization of arterial blood and f is the CBF in $ml_{\mathrm{blood}}/ml_{\mathrm{tissue}}/s$. $c\left(t\right)$, $r\left(t\right)$, and $m\left(t\right)$ denote the delivery function, residue function, and magnetization relaxation function, respectively. The ∗ denotes convolution operation. For pseudo‐continuous ASL (pCASL), these functions take the form of:

$$c\left(t\right)=\left\{\begin{array}{cc} 0 & 0<t<\Delta t\\ \alpha e^{‐\Delta t/T_{1b}} & \Delta t<t<\tau +\Delta t\\ 0 & \tau +\Delta t<t \end{array}\right.$$

$$r\left(t\right)=e^{‐ft/\lambda }$$

$$m\left(t\right)=e^{‐t/T_{1t}},$$

where α is the labeling efficiency, Δ*t* is the ATT of tissue, τ is the label duration, $T_{1b}$ and $T_{1t}$ are the longitudinal relaxation constants of arterial blood and tissue, respectively, and λ is the blood‐tissue partition coefficient of water.

The form of the difference signal of the macrovascular compartment^8^ is related to the delivery function $c\left(t\right)$ but has its own transit time Δ*t_a_*:

$$\Delta M_{a}\left(t\right)=\left\{\begin{array}{cc} 0 & t<\Delta t_{a}\\ 2\alpha M_{0B}e^{‐\Delta t_{a}/T_{1b}}\cdot aBV & \Delta t_{a}<t<\Delta t_{a}+\tau \\ 0 & \Delta t+\tau <t \end{array}\right.$$

where aBV is the arterial blood volume fraction in percentage. The total signal in a voxel is the sum of its tissue compartment and macrovascular compartment, that is, $\Delta M\left(t\right)=\Delta M_{t}\left(t\right)+\Delta M_{a}\left(t\right)$.

Flow dispersion can be accounted for by convolving the delivery function $c\left(t\right)$ with a dispersion kernel $k\left(t\right)$ to change its shape. Therefore,

$$c^{\prime }\left(t\right)=c\left(t\right)*k\left(t\right).$$

Here, we chose a Gamma dispersion kernel for its physiologically plausible shape and relatively low computational complexity^12^:

$$k\left(t\right)=\frac{s^{1+sp}}{\Gamma \left(1+sp\right)}t^{\mathrm{sp}}e^{‐st}$$

where $\Gamma \left(x\right)$ is the Gamma function, and s, p depicts the “sharpness” and time‐to‐peak characteristics of the dispersion kernel. The lower the value of s, the less sharp the Gamma kernel is, and the higher degree of dispersion the kernel adds to the signal. In this study, we used the same dispersion parameters for both tissue and macrovascular components.

By switching whether dispersion or macrovascular contamination is included, four types of signals were simulated: no dispersion no macrovascular contamination (D‐M‐), no dispersion with macrovascular contamination (D‐M+), with dispersion no macrovascular contamination (D+M‐), and with dispersion with macrovascular contamination (D+M+).

### 2.2 Simulation experiments

Four different PLD protocols from Woods et al were investigated: single‐PLD, reference multi‐PLD, CBF‐ATTopt, and CBFopt.^7^ These protocols were optimized for CBF and/or ATT accuracy for an ATT range of 0.5 s to 1.8 s in the absence of dispersion and macrovascular signal. Table 1 lists the PLDs and number of averages for each protocol obtained by optimization. For each protocol, pCASL signals were simulated in MATLAB (The Mathworks, Natick, MA) using the four models in the previous section. Two factors were varied in simulation: ATTs from 0.5 s to 3.0 s with 0.05 s interval, which includes ATT values much longer than those which had been optimized for, and an aBV range from 0 to 2% with 0.05% interval. To test the effects of flow dispersion, simulations were performed three times based on different values of s (and a constant $p_{0}=0.17s$) in the Gamma kernel: $s_{0}=1/0.13$ s^−1^, $s_{0}/2$, and $s_{0}/4$, each with a higher degree of flow dispersion. Supporting Information Figure S1, which is available online, illustrates the effect of different dispersion kernels on both tissue and macrovascular signals. Gaussian white noise was then added to all signals, with its SD defined by the maximum signal intensity sampled across all PLDs in a typical condition (ATT = 1.4 s, aBV = 0.2%) over a signal‐to‐noise ratio (SNR) of 9.37 under common MR acquisition environment,^13^ that is, $SD_{\mathrm{noise}}=\Delta M_{\text{samp\_max}}/SNR$. Each condition was repeated for 2000 times. See Table 2 for all simulation parameters. For the purposes of simplification in this study, macrovascular ATT was fixed relative to tissue ATT by 0.5 s, this implicitly assumes that the mechanism that prolongs the ATT (eg, pathology in the feeding arteries) affects both the macrovascular and tissue blood supply by the same amount, but the reality in vivo may be more complex than this in practice.

**TABLE 1:** Protocol timings from Woods et al^7^

Protocol	Post‐labeling delays (s)	PLDs (N)	Averages (N)
Single‐PLD	1.8	1	33
Reference multi‐PLD	0.25, 0.5, 0.75, 1, 1.25, 1.5	6	7
CBF‐ATTopt	0.2, 0.2, 0.225, 0.3, 0.375, 0.45, 0.5, 0.55, 0.6, 0.6, 0.625, 0.625, 0.65, 0.65, 0.675, 0.675, 0.7, 0.7, 0.7, 0.7, 1.25, 1.275, 1.3, 1.35, 1.375, 1.4, 1.425, 1.425, 1.475, 1.5, 1.675, 1.75, 1.8, 1.825, 1.85, 1.875, 1.9, 1.925, 1.95, 1.975	40	1
CBFopt	0.2, 0.7, 0.825, 1, 1.125, 1.25, 1.325, 1.4, 1.475, 1.55, 1.625, 1.675, 1.7, 1.725, 1.75, 1.775, 1.8, 1.825, 1.85, 1.85, 1.875, 1.9, 1.925, 1.925, 1.95, 1.975, 1.975, 2, 2.025, 2.025, 2.05, 2.075, 2.075	34	1

**TABLE 2:** Parameters used for simulations

Parameter	Value
Cerebral blood flow (f)	60 ml/100 g/min
Blood‐tissue partition coefficient of water (λ)	0.9 ml/g
T1 of arterial blood ($T_{1b}$)	1.65 s
T1 of tissue ($T_{1t}$)	1.3 s
Labeling efficiency (α)	0.85
Label duration (τ)	1.4 s
ATT of macrovascular compartment ($\Delta t_{a}$)	Δt−0.5 s
Arterial blood volume (aBV) when varying tissue ATT	0.2%
Tissue ATT (Δt) when varying aBV	1.4 s
Sharpness of Gamma dispersion kernel ($s_{0}$)	1/0.13 s^−1^
Time‐to‐peak of Gamma dispersion kernel ($p_{0}$)	0.17 s

Noisy signals for each protocol were then fit with different kinetic models to obtain CBF and ATT (only for multi‐PLD protocols) estimates and errors using the BASIL toolkit from the Oxford Centre for Functional MRI of the Brain (FMRIB)'s software library (FSL),^14^ which uses a variational Bayesian inference method with estimation priors applied to each parameter. See Supporting Information Table S1 for a list of parameter priors used. All signals were fit with the general kinetic model (denoted gkm). D‐M+, D+M‐, and D+M+ data were also fitted with extended kinetic models by accounting for macrovascular contamination (denoted gkm+mvc), dispersion (denoted gkm+disp), or both (denoted gkm+disp+mvc). For the full list of models fit used, see Table 3. Apart from estimating for CBF and ATT in gkm, aBV and $\Delta t_{a}$ were estimated in gkm+mvc and gkm+disp+mvc, while s and p were estimated in gkm+disp and gkm+disp+mvc. Other parameters assumed the values of the priors in Supporting Information Table S1 when an estimation was not possible. For model fitting of the single‐PLD protocol in simulation, we assumed an ATT of 1.25 s.^7^ CBF and ATT estimation error using each model was calculated by “(estimated value − ground truth value)/ground truth value * 100.” Results were compared both across models and across protocols. As a measure of each protocol’s sensitivity to macrovascular contamination, we calculated the slopes of the CBF and ATT estimation error line of best fit when aBV > 0.5% for D‐M+ signals fitted with gkm and gkm+mvc.

**TABLE 3:** Models used for fitting each signal

Signal	Models used for fitting
No dispersion no macrovascular contamination (D‐M‐)	general kinetic model (gkm)
No dispersion with macrovascular contamination (D‐M+)	gkm, gkm+mvc
With dispersion no macrovascular contamination (D+M‐)	gkm, gkm+disp
With dispersion with macrovascular contamination (D+M+)	gkm, gkm+mvc, gkm+disp, gkm+disp+mvc

### 2.3 In vivo experiments

A dataset from seven healthy subjects (three female, 23‐27 y old) from Woods et al was retrospectively analyzed to examine the consistency between the results of simulations and in vivo acquisition.^7^ The pCASL imaging parameters were: 2D‐EPI, five slices, voxel size = 3.4 × 3.4 × 5 mm^3^, 1.4 s label duration, and flow crusher gradients with a cutoff velocity of 4 cm/s. A full list of imaging parameters and pre‐processing methods can be found in Woods et al.^7^ Gray matter (GM) masks were obtained from the T1 structural image using the FAST tool from FSL.^15^ CBF and ATT estimates were obtained by fitting the data of each protocol with the four models. The estimates were then binned and averaged with 0.05 s ATT or 0.05% aBV interval across all subjects, so as to match the sampling points in simulation.

Another four sets of CBF and ATT estimates were obtained by fitting the four models (gkm, gkm+disp, gkm+mvc, gkm+disp+mvc) to the combined data from all four protocols, giving an equal weighting to each protocol. Voxels were excluded if the CBF and ATT estimated by gkm from the combined data had SDs higher than 15 ml/100 g/min and 1.0 s, respectively, and were restricted by the parameter range of interest (0.5 s < ATT < 3.0 s, 0.0 < aBV < 2.0%). These sets of estimates were used in place of ground‐truth estimates in calculating estimation errors of each protocol, since there was no independent gold‐standard protocol or universal ground‐truth for the in vivo data. For example, the CBF error of Reference Multi‐PLD estimated by gkm with respect to the CBF estimated by gkm+mvc from the combined data was calculated by

$$\text{CBF}\;\text{Error}{\%}_{\text{Ref-multi},\text{gkm}}^{\text{gkm}+\text{mvc}}=\frac{{\text{CBF}}_{\text{Ref - multi},\;\text{gkm}}‐{\text{CBF}}_{\text{combined},\;\text{gkm}+\text{mvc}}}{{\text{CBF}}_{\text{combined},\;\text{gkm}+\text{mvc}}}\times 100$$

The resulting estimation errors were compared to the errors in the equivalent simulation study. For example, the above CBF estimation error was compared to the CBF error of Reference Multi‐PLD using D‐M+ signals fit by gkm. This allowed an assessment of whether differences seen using the gkm on real data when compared to using a more sophisticated model matched with the errors seen in simulation when dispersion and/or macrovascular contamination were included in the signal.

Similar to the sensitivity to MVC measure obtained in simulation, slopes of CBF and ATT estimation errors fit with gkm and gkm+mvc (with respect to CBF_combined, gkm+mvc_ and ATT_combined, gkm+mvc_) were generated by linear regression when aBV > 0.5%. All statistical tests were performed using two‐sample t‐tests (one‐tailed, with Bonferroni correction for multiple comparisons, *P*‐value threshold at .05) between estimations by different protocols.

## 3 RESULTS

**FIGURE 1:**
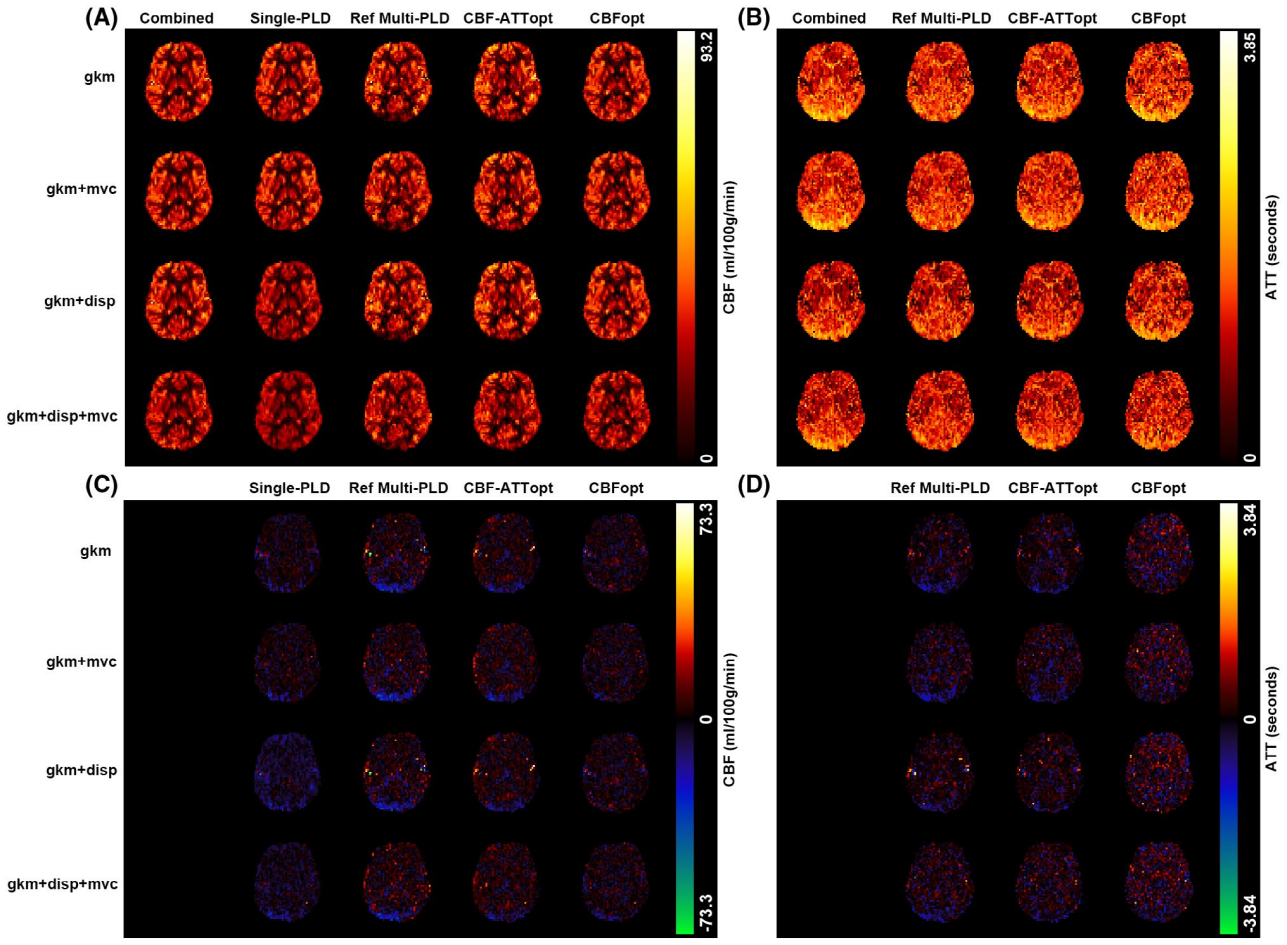
Representative whole‐brain CBF and ATT estimation maps and absolute error maps (protocols − combined) for the four protocols tested and combined data estimates (by column), and for the four estimation models (by row). The maps show one axial slice from a single subject. A, CBF estimation map. B, ATT estimation map. C, CBF absolute error map. D, ATT absolute error map

Using the four models to fit in vivo data of different protocols, whole brain and segmented GM CBF and ATT maps were obtained. Across all subjects, 1216 voxels were identified as having prolonged ATT (ATT > 1.8 s, 6.19% of all GM voxels), and 1268 voxels as having high macrovascular signal (aBV > 0.25%, 6.46% of all GM voxels). Representative whole‐brain CBF and ATT estimation maps and absolute error maps (protocols − combined) from one subject are shown in Figure 1. Reasonably good visual agreement was found between different protocols and fitting models. Evidence of macrovascular contamination could be seen in some cortical voxels in reference multi‐PLD and CBF‐ATTopt, which appeared as higher CBF estimation. CBFopt appeared to be in best agreement with the CBF estimation from the combined data among the three multi‐PLD protocols.

**FIGURE 2:**
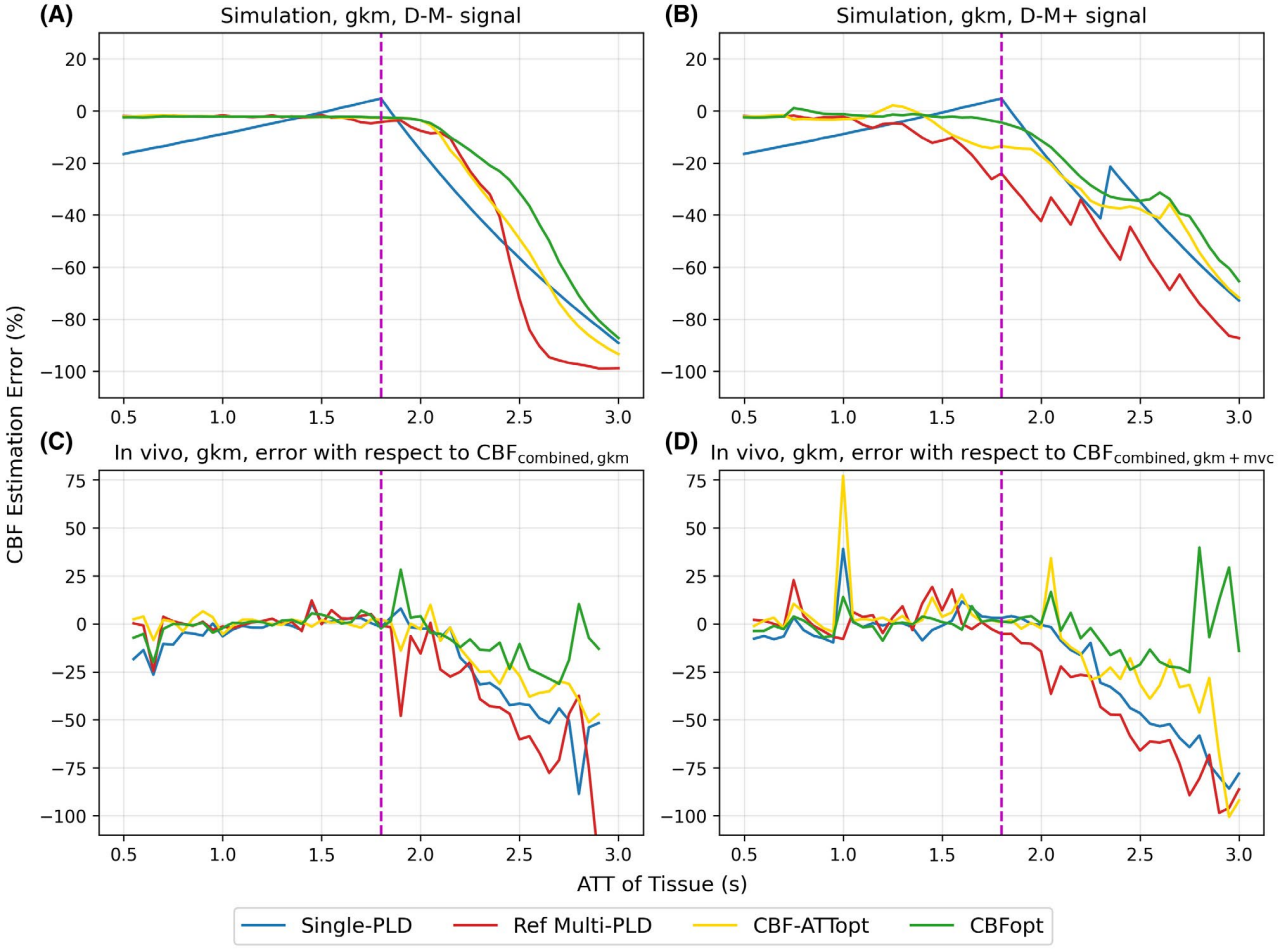
CBF estimation mean errors for the four protocols fit with gkm. A, Simulation data using D‐M‐. B, Simulation data using D‐M+, with aBV = 0.2%. C, In vivo estimation error with respect to CBF_combined, gkm_. D, In vivo estimation error with respect to CBF_combined, gkm+mvc_. The dashed magenta line indicates the upper limit of the range of ATT that CBF‐ATTopt and CBFopt were optimized for

Figure 2 shows CBF estimation errors for the different protocols with and without macrovascular contamination both in simulation and in vivo using gkm as the fitting model. The general trends were consistent between simulation and in vivo data. All three multi‐PLD protocols performed well within a normal range of ATT (ATT < 1.8 s), with only Ref Multi‐PLD protocol exhibiting substantial error from macrovascular contamination within that range. In the absence of MVC, multi‐PLD protocols could retain CBF accuracy beyond an ATT of 2.0 s up to 2.5 s for CBFopt (Figure 2A,C). No protocol had a consistently better performance than others when macrovascular contamination was present over the wider ATT range (Figure 2B,D). ATT estimation errors of the three multi‐PLD protocols under the same conditions are shown in Supporting Information Figure S2. ATT was underestimated by all three protocols both in simulation and in vivo when ATT was prolonged. Despite having higher ATT estimation errors at short ATTs, CBFopt had the lowest ATT errors among all protocols at prolonged ATTs.

**FIGURE 3:**
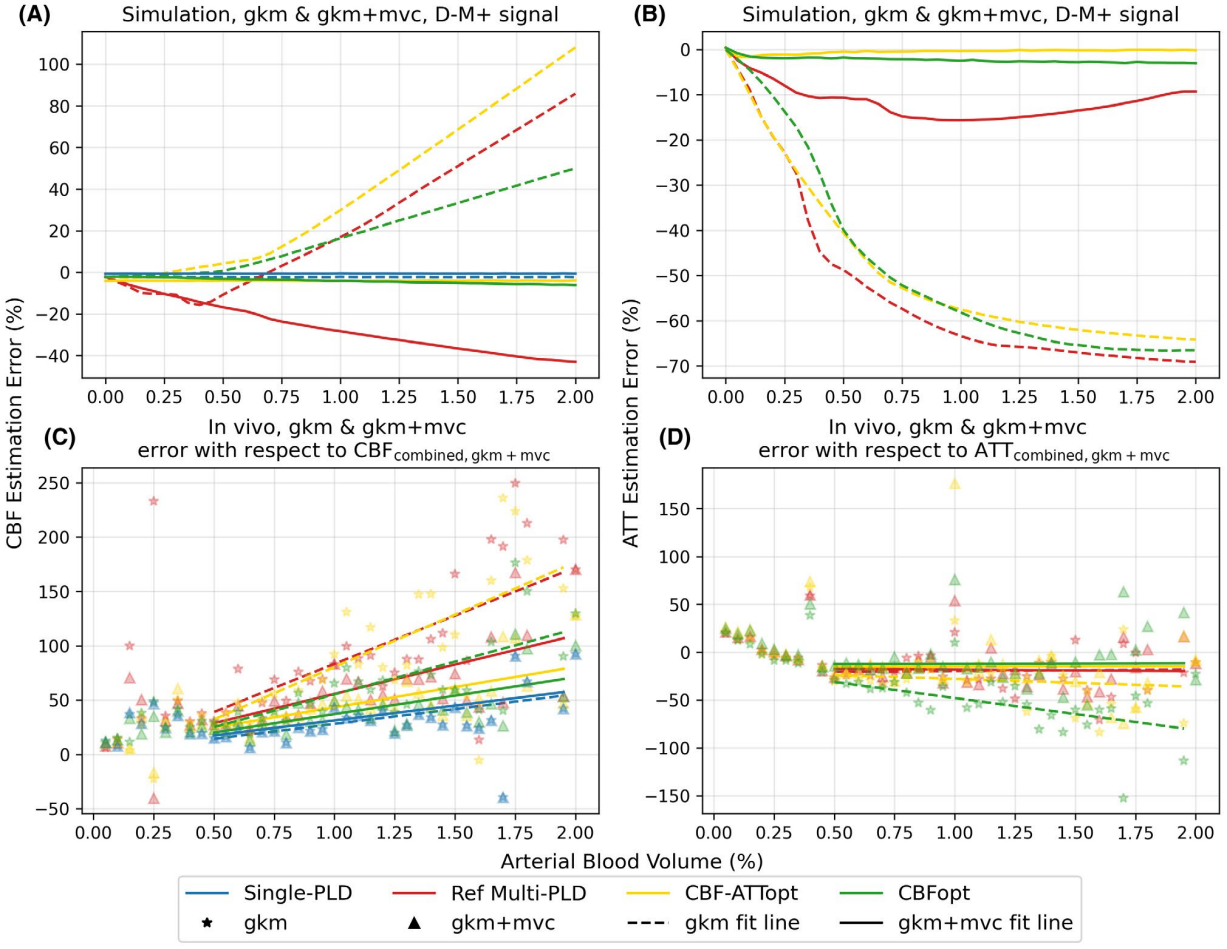
CBF and ATT estimation errors for the four protocols fitted with gkm and gkm+mvc over a range of aBV values. ATT was held constant at 1.4 s in simulation across all aBVs. A, CBF errors of simulation data on D‐M+ signals. B, ATT errors of simulation data on D‐M+ signals. C, CBF errors of in vivo data. D, ATT errors of in vivo data. In vivo estimation errors were calculated with respect to CBF_combined, gkm+mvc_ and ATT_combined, gkm+mvc_. The star and triangle markers in (C) and (D) represent the mean errors at each binned value of aBV fit by gkm and gkm+mvc, respectively, while the dashed and solid lines represent the linear fit of errors when aBV > 0.5% by gkm and gkm+mvc, respectively

**FIGURE 4:**
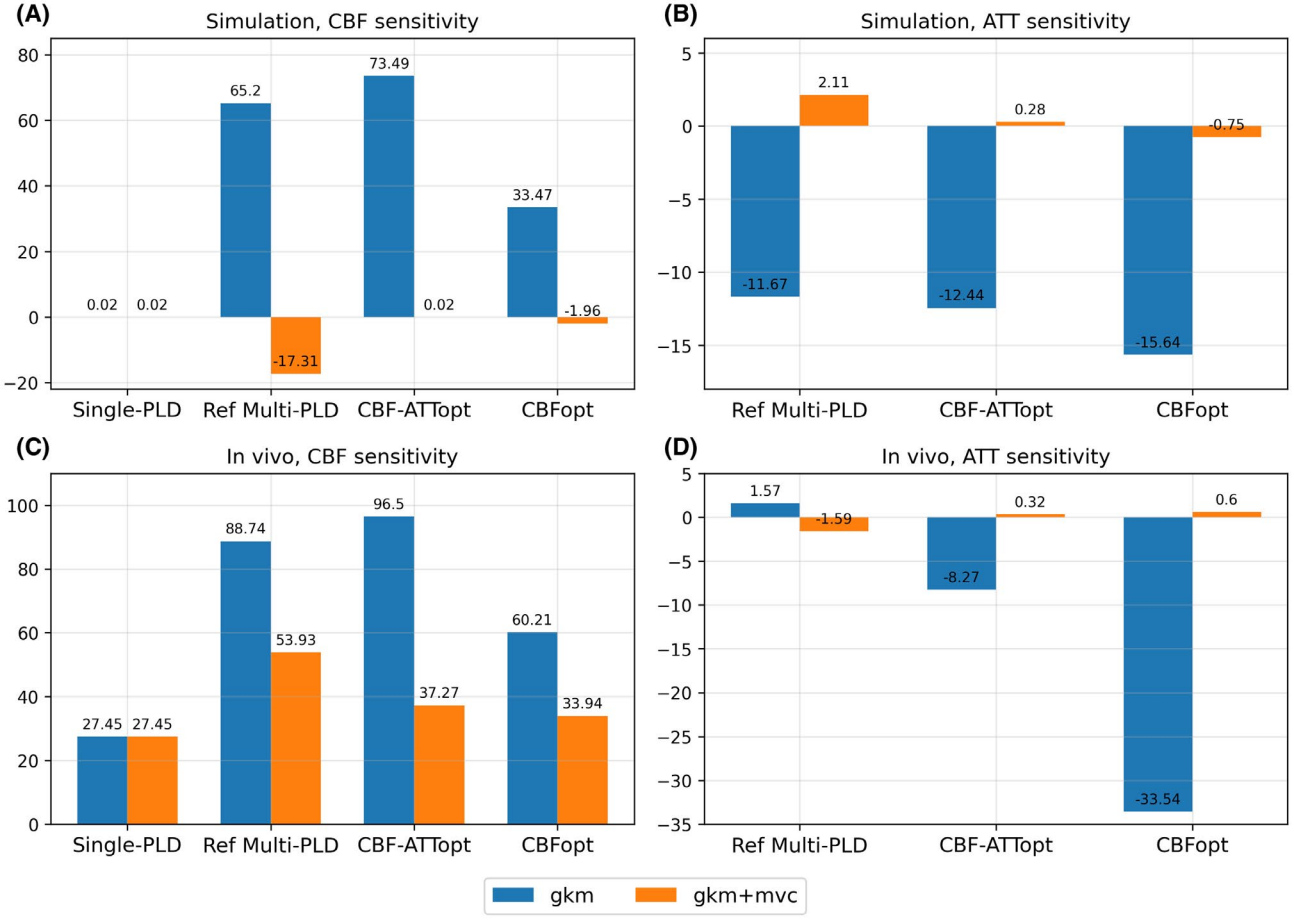
A‐D, CBF and ATT sensitivity (unit: % error per 1% aBV) to macrovascular contamination for the four protocols with conditions in Figure 3. Sensitivity values represent the slopes of the lines of best fit when aBV > 0.5%

Figure 3 shows CBF and ATT estimation errors over a range of aBV both in simulation and in vivo using gkm and gkm+mvc as fitting models. The bar chart in Figure 4 shows the sensitivity values to macrovascular contamination, measured by the slope of error against aBV. When aBV was varied, all three multi‐PLD protocols were sensitive to macrovascular contamination when fitted with gkm, overestimating CBF and underestimating ATT (see dashed lines in Figure 3A,B and star marks in Figure 3C,D). For CBF estimation in simulation, CBFopt had the smallest sensitivity value to macrovascular contamination (33.47% error per 1% aBV) among multi‐PLD protocols using gkm. For ATT estimation, the three multi‐PLD protocols all significantly underestimated ATT, but were not significantly different from each other. Despite having different ground truth CBF values from simulation, similar relations were seen using in vivo data, where CBF sensitivity to macrovascular contamination of CBFopt (60.21% error per 1% aBV) was much smaller than the reference multi‐PLD (88.73% error per 1% aBV) and CBF‐ATTopt (96.5% error per 1% aBV). By fitting the signal with an extended kinetic model gkm+mvc, all multi‐PLD protocols showed a notable decrease in CBF and ATT estimation error in simulation (solid lines in Figure 3A,B) and in vivo (triangle marks in Figure 3C,D). Absolute CBF and ATT sensitivity values to macrovascular contamination were all lowered except for the absolute ATT sensitivity for the reference multi‐PLD protocol with in vivo data (Figure 4).

**FIGURE 5:**
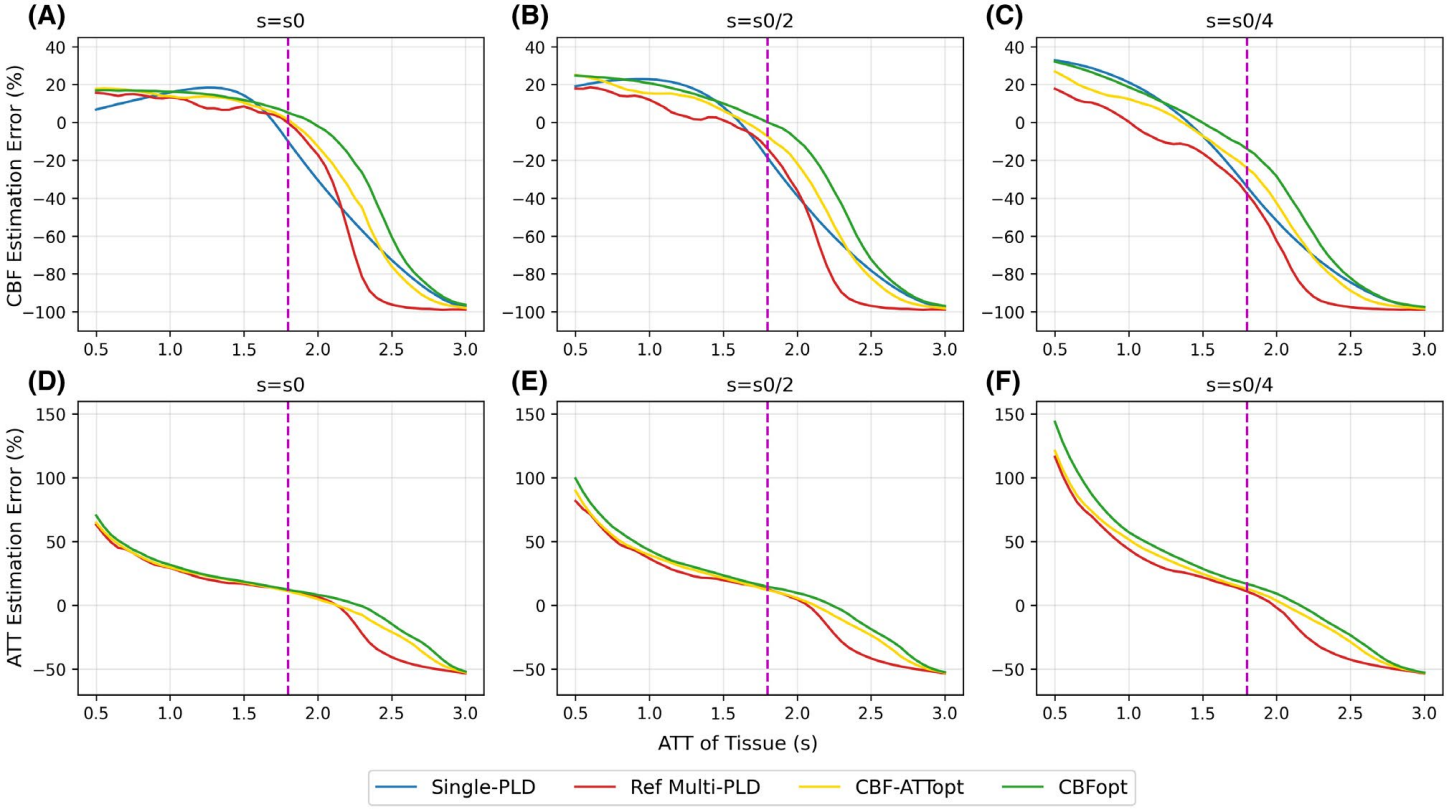
CBF and ATT estimation fitted with gkm over a PAR on D+M‐ signals with different degrees of dispersion. A,D, $s=s_{0}$, low degree of dispersion. B,E, $s=s_{0}/2$, moderate degree of dispersion. C,F,: $s=s_{0}/4$, high degree of dispersion. The dashed magenta line indicates the upper limit of the range of ATT that CBF‐ATTopt and CBFopt were optimized for

When the degree of dispersion was varied in D+M‐ signals in simulation but not accounted for in the model fitting, as shown in Figure 5, both CBF and ATT were overestimated at shorter ATT and CBF was underestimated at prolonged ATT by all protocols. The CBF and ATT overestimation errors increased with a higher degree of dispersion, with the highest increase by CBFopt being 7.82% and 15.23% in CBF estimation and 29.07% and 73.42% in ATT estimation when $s=s_{0}/2$ and $s=s_{0}/4$, respectively. The largest error of CBF underestimations all reached nearly −100% at the longest ATT, when the protocols could hardly detect meaningful signals due to the prolonged arrival time. When dispersion was accounted for in the model fitting by gkm+disp, which is shown in Supporting Information Figure S3, reference multi‐PLD could retain CBF estimation accuracy up to 2.0 s, while optimized protocols up to 2.5 s. ATT estimation errors were significantly reduced when ATT < 1.8 s. Beyond the ATT range of 2.5 s, however, the three protocols did not show consistent increase or reduction of CBF and ATT errors.

## 4 DISCUSSION

In this work, we have explored the performance of several ASL imaging protocols in CBF and ATT estimation under the effects of macrovascular signal contamination, flow dispersion, and prolonged ATT.

### 4.1 Macrovascular contamination

The single PLD value recommended by the ASL consensus paper^3^ and tested in this study is 1800 ms, a trade‐off between incomplete arrival of blood to the tissue voxels and a loss of ASL signal due to T_1_ relaxation. If the PLD is too short, part of the labeled blood may remain in the macrovasculature, leading to false interpretation of tissue perfusion by macrovascular signals. This is particularly problematic in multi‐PLD ASL, since short PLDs are often used to capture the arrival time information. When macrovascular contamination was included (Figure 3), CBF was overestimated and ATT was underestimated across all protocols at high aBV when using gkm, that is, not accounting for macrovascular contamination in the analysis. CBFopt had the least sensitivity to MVC of the multi‐PLD protocols when gkm was used for analysis, as indicated by the smallest slope value against an increasing aBV, most probably because it mainly includes long PLDs within the sampling schedule. The three multi‐PLD protocols yielded similar but high ATT estimation errors, significantly underestimating the ATT when no macrovascular compartment was included in the model. This would be consistent with the model treating the macrovascular signal as tissue perfusion with a shorter ATT. When the extended model gkm+mvc was used, that is, the analysis explicitly attempted to correct for macrovascular contamination, all protocols showed a large improvement in CBF and ATT estimation. This was seen by a decrease of almost all CBF and ATT sensitivity values by multi‐PLD protocols using gkm+mvc than the original gkm in Figure 4. CBF‐ATTopt showed significantly lower error in estimating arterial blood volume than Reference multi‐PLD and CBFopt using gkm+mvc, shown by Supporting Information Figure S4. This benefit could be due to the short ATTs within the CBF‐ATTopt protocol.

When MVC was present in a PAR, shown in Figure 2B and Figure S2B, no protocol exhibited consistently better performance than others in CBF and ATT estimation. It is worth noting that, because flow crusher gradients were used to eliminate most of the macrovascular signal in the in vivo data, estimation errors with respect to CBF_combined, gkm+mvc_ (Figure 2D, Figure S2D) was not the in vivo equivalent of estimation using simulated D‐M+ signals. A more direct comparison would be to use gkm+mvc to fit D‐M‐ signals, which is presented in Supporting Information Figure S5. For both CBF and ATT estimation, fitting with gkm+mvc led to higher errors when ATT < 1.3 s, while the errors remained comparable to fitting with gkm when ATT > 1.3 s, with CBFopt achieving smaller estimation SD. This, together with the fact that the in vivo results using CBF_combined, gkm+mvc_ in error calculation were not substantially different from the results using CBF_combined, gkm_ (Figure 2D,C), suggests that explicitly including a macrovascular compartment in the model used for analysis could be a successful strategy for controling for MVC, without necessarily having the knowledge of whether or not MVC was present.

The in vivo data used in this study included flow crusher gradients, as the study from which the data originated sought to test protocols without any macrovascular signal.^7^ Bipolar flow crusher gradients can be used to eliminate flowing labeled spins in one direction above a cutoff velocity. However, the macrovascular signals perpendicular to the gradient direction are unaffected and present in the image. Thus, in practice, macrovascular contamination remains, as has been seen previously,^8^ and has been exploited here to examine the sensitivity to macrovascular contamination. In general, the ASL white paper discouraged the use of flow crusher gradients,^3^ concerning that important clinical information might be removed, but leading to more prominent macrovascular signal contamination. Therefore, we would recommend the use of the two‐compartment model in multi‐PLD acquisitions to control, as well as potentially estimate for, the effect of MVC.

### 4.2 Dispersion effects

We investigated the effects of flow dispersion by varying the sharpness parameter s in the Gamma kernel to control the degree of dispersion. All protocols showed overestimation of CBF and ATT at short ATT and underestimation of CBF and ATT at prolonged ATT. This CBF overestimation is contrary to previous pulsed‐ASL findings,^9^, ^16^, ^17^ in which dispersion effects were reported to cause an underestimation of CBF. A possible explanation is that, while the dispersion kernel attenuates the peak of the signal curve, the protocols examined in this study were mainly sampling the trailing edge of the curve when ATT was short, where dispersion increases the signal magnitudes compared to the non‐dispersed case using the dispersion model implemented here. Across the three dispersion parameters tested, $s=s_{0}/4$ generated the greatest dispersion which in turn led to the highest error of CBF and ATT overestimation at short ATT, shown by Figure 5C,F. However, the bias due to dispersion was pretty consistent between different protocols. This suggests that, while flow dispersion does influence CBF and ATT estimation, the presence of dispersion would not alter the appropriateness of the existing optimal sampling framework, and our choice of an optimal protocol *per se* would not lead to particular egregious errors.

In this study, we chose a Gamma‐shaped dispersion kernel, which has a relative advantage in physiological plausibility and computational simplicity.^12^ Other shapes of dispersion kernels or vascular transport functions (VTFs) have also been proposed, such as the Gaussian kernel^16^, ^18^ and the delay‐dependent exponential kernel used in dynamic susceptibility contrast (DSC) imaging.^19^ Furthermore, there is debate as to whether one dispersion kernel independent to spatial location could accurately describe the profile of flow dispersion within every voxel. For instance, the macrovascular component was seen to show a higher sensitivity to the choice of dispersion kernel than the tissue component.^12^ Future work could look into the effects of having different levels of dispersion between tissue and macrovascular compartments. In addition, the effects of dispersion could be controlled by determining an AIF that is upstream to the voxel, that is, a local AIF. Methods, such as subtraction of signals with and without flow crusher gradients^20^ or using independent component analysis,^21^ have been developed to obtain a local AIF, thus reducing the errors arising from dispersion effects.

### 4.3 Prolonged ATT

The ATT range used in the optimization of CBF‐ATTopt and CBFopt was [0.5 s, 1.8 s]. In this study, we tested the performance of the protocols with an extended ATT range [0.5 s, 3.0 s] to reflect potential unexpected deviations from this narrow range in practice, although for application in cohorts where longer ATTs are expected, the optimization range should be adjusted (eg, see Woods et al^7^ and Supporting Information Table S1).

For CBF estimation, all four protocols underestimated CBF at prolonged ATT, as expected. We could see from Figure 2 that the single‐PLD protocol was much more sensitive to the change of tissue ATT than the multi‐PLD protocols in the normal ATT range. Although the consensus paper recommended the use of a 1800 ms single‐PLD protocol for young and healthy adults, and 2000 ms single‐PLD for elderly subjects or pathological conditions,^3^ our results showed a worse performance of the single‐PLD protocol than multi‐PLD protocols under the tested SNR. In keeping with the study of Woods et al,^7^ the estimation by the optimized multi‐PLD protocols, CBF‐ATTopt and CBFopt, was quite satisfactory until tissue ATT was out of their initial optimized range. CBFopt and CBF‐ATTopt remained reasonably accurate up to ATT = 2.5 s for CBF and ATT estimation, respectively (Figure 2A and Supporting Information Figure S2A). The standard deviations of CBF and ATT estimation errors in simulation are shown in Supporting Information Figure S6. Estimation SDs by gkm+mvc were significantly smaller than those by gkm in the PAR. This might be because the presence of the extra signal from the macrovascular component gives a higher SNR, leading to a more consistent parameter fitting. In the in vivo results, CBFopt had significantly lower absolute errors in CBF estimation than all other protocols in the extended ATT range, whether the errors were calculated with respect to CBF_combined, gkm_ or CBF_combined, gkm+mvc_ (average reduction of 4.96% or 9.45% CBF estimation error compared to CBF‐ATTopt, respectively), and without having significant differences in ATT errors compared to CBF‐ATTopt. In accordance with the lowest sensitivity value to MVC of CBFopt; therefore, we argue that CBFopt is the least sensitive protocol to MVC and unexpectedly prolonged ATT for CBF estimation, while maintaining reasonably good performance in estimating ATT. We may further conclude that the optimization framework can still be used where some prolonged ATTs (up to 2.5 s) might be expected, particularly where the majority of the voxels are likely to fall within the optimal range. Beyond this ATT range, the protocols would become overly sensitive to the long ATTs, and the optimization range should be reconsidered.

### 4.4 Results from 3D‐readout protocols

To investigate further the effects of using a protocol outside of its optimized range, more simulations were performed using the optimal protocols designed for segmented 3D‐readout,^7^ which are listed in Supporting Information Table S2. These two sets of protocols were optimized for a normal ATT range (NAR) [0.5 s, 2.0 s] and a PAR [1.0 s, 3.0 s], and were both tested under the same simulation conditions across the aBV range [0.0, 2.0%] and the ATT range [0.5 s, 3.0 s] as the 2D‐readout case.

CBF and ATT estimation errors across the aBV range can be found in Supporting information Figure S7. PAR protocols appeared to be more predictable than NAR protocols, giving near‐linear sensitivity to aBV in both estimations when aBV > 1.25% (Supporting Information Figure S7B). Furthermore, PAR protocols produced more consistent results when using gkm+mvc, clearly reducing the estimation error, whereas in NAR protocols the results were more variable. CBF and ATT estimation errors across the PAR can be found in Supporting Information Figure S8. Within the overlapping ATT range of [1.0 s, 2.0 s], there were similar CBF and ATT estimation errors between NAR and PAR versions of CBF‐ATTopt and CBFopt. However, NAR protocols had a consistently lower standard deviation than PAR protocols for CBF‐ATTopt and CBFopt in this range. Expectedly, NAR protocols had better performance when ATT < 1.0 s, while PAR protocols did better when ATT > 2.0 s. This indicates that optimized protocols would evidently yield higher accuracy within their optimized range, but could only work sub‐optimally outside this range. Choosing the appropriate optimized protocol, thus, is critical in controlling estimation errors. In the meantime, if prior knowledge of the ATT profile of the dataset is provided, a non‐uniform ATT distribution can be used in the optimization process to give different weights to the ATTs within the optimized range.

### 4.5 Limitations

This study has a few limitations. First, the results are based on the estimation from the extended kinetic model that assumes a specific shape of dispersion kernel and macrovascular AIF. Meanwhile, other artifacts might also be present in real data, further confounding the accurate quantification of perfusion and ATT. For example, T2* differences between capillary and tissue might cause an underestimation of CBF using the one‐compartment model; partial volume effects in which different composites of the brain tissue share the same voxel will underestimate perfusion in the gray matter and overestimate perfusion in the white matter. Second, assumptions have been made at various stages in simulation that might be violated in vivo. For example, the difference between bolus arrival times of the macrovascular signal and the tissue signal is assumed to remain a constant of 0.5 s in this study. Further research is needed to investigate how this assumption might affect the outcome. It is also worth noting that the improvement of CBF estimation seen in simulated data using gkm+mvc should not be over‐interpreted as implying that the observed benefit will necessarily be achieved in real data since these are idealized experiments in which the same model is used to fit the model‐generated data.

Third, the optimized protocols so far have only been applied to a young and healthy population, who typically have the ATTs in the optimized range. This work did preliminary analyses on a small dataset that included limited voxels that had prolonged ATT. Future work could explicitly focus on elderly or pathological groups, investigate the effects of these protocols on long ATTs, and compare them to appropriately optimized protocols for these groups. This could help establish the importance of protocol optimization for each sub‐population due to their varying ATT ranges.

## 5 CONCLUSIONS

In conclusion, we have examined four ASL protocols under the effects of dispersion and macrovascular contamination over a PAR. Explicitly including a macrovascular component in the kinetic model was shown to be a feasible approach in controlling for MVC. Furthermore, we argue that, among all protocols, CBFopt was the least sensitive protocol to MVC and unexpectedly prolonged ATT for CBF estimation while maintaining reasonably good performance in estimating ATT.

## Supporting information


**FIGURE S1** The effect of different dispersion kernels to the tissue (solid lines) and macrovascular signals (dashed lines). A smaller parameter s indicates a higher level of flow dispersion. Parameters used in this simulation: tissue ATT = 1.4s, macrovascular ATT = 0.9s
**FIGURE S2** ATT estimation errors for the 3 multi‐PLD protocols fitted with gkm over a prolonged ATT range. (A): simulation data using D‐M‐; (B): simulation data using D‐M+; (C): in vivo estimation error with respect to ATT_combined, gkm_; (D): in vivo estimation error with respect to ATT_combined, gkm+mvc_. The dashed magenta line indicates the upper limit of the range of ATT that CBF‐ATTopt and CBFopt was optimised for
**FIGURE S3** Simulation CBF and ATT estimation errors for the 4 protocols fit with gkm or gkm+disp using D+M‐ signals (kernel sharpness $s=s_{0}$) over a prolonged ATT range. (A): CBF errors fit with gkm; (B): CBF errors fit with gkm+disp; (C): ATT errors fit with gkm; (D): ATT errors fit with gkm+disp
**FIGURE S4** Arterial blood volume (aBV) estimation means and standard deviations for the 3 multi‐PLD protocols fitted with gkm+mvc using D‐M+ signals. The dashed magenta line indicates identity
**FIGURE S5** Simulation CBF and ATT estimation error means and standard deviations for the 4 protocols fitted with gkm or gkm+mvc using D‐M‐ signals over a prolonged ATT range
**FIGURE S6** Simulation CBF and ATT estimation error standard deviations for the 4 protocols fitted with gkm over a prolonged ATT range. (A): CBF error std using D‐M‐; (B): CBF error std using D‐M+; (C): ATT error std using D‐M‐; (D): ATT error std using D‐M+. The dashed magenta line indicates the upper limit of the range of ATT that CBF‐ATTopt and CBFopt was optimised for
**FIGURE S7** Simulation CBF and ATT estimation errors for the 3 normal‐range protocols and 3 prolonged‐range protocols fit with gkm over a range of aBV using D‐M+ signals. ATT was held constant at 1.4s in simulation across all aBVs. (A): CBF errors of normal‐range protocols; (B): CBF errors of prolonged‐range protocols; (C) ATT errors of normal‐range protocols; (D): ATT errors of prolonged‐range protocols
**FIGURE S8** Simulation CBF and ATT estimation error means and standard deviations for the 3 normal‐ATT‐range (NAR) protocols and 3 prolonged‐ATT‐range (PAR) protocols fitted with gkm over a prolonged ATT range using D‐M‐ signals. (A): CBF errors of NAR protocols; (B): CBF errors of PAR protocols; (C) CBF errors std of NAR & PAR protocols; (D) ATT errors of NAR protocols; (E): ATT errors of PAR protocols; (F): ATT errors std of NAR & PAR protocols
**TABLE S1** Specifications of the estimation priors (as mean and standard deviation by a normal distribution) used in the variational Bayesian inference method for simulation experiments
**TABLE S2** Protocol timings of 3D readout from Woods *et al^7^*. Two sets of protocols were developed, one for a normal range of 0.5≤ATT≤2.0s, the other one for a prolonged range of 1.0≤ATT≤3.0s
