# Supplementary material for: Examination of optimized protocols for pCASL: Sensitivity to macrovascular contamination, flow dispersion, and prolonged arterial transit time
Source: Magn Reson Med. 2021 May 19;86(4):2208–19. doi: 10.1002/mrm.28839 (PMC8581991; doi:10.1002/mrm.28839)
Supplement: Supplementary file 1 — FIGURE S1 The effect of different dispersion kernels to the tissue (solid lines) and macrovascular signals (dashed lines). A smaller parameter s indicates a higher level of flow dispersion. Parameters used in this simulation: tissue ATT = 1.4s, macrovascular ATT = 0.9s FIGURE S2 ATT estimation errors for the 3 multi‐PLD protocols fitted with gkm over a prolonged ATT range. (A): simulation data using D‐M‐; (B): simulation data using D‐M+; (C): in vivo estimation error with respect to ATTcombined, gkm; (D): in vivo estimation error with respect to ATTcombined, gkm+mvc. The dashed magenta line indicates the upper limit of the range of ATT that CBF‐ATTopt and CBFopt was optimised for FIGURE S3 Simulation CBF and ATT estimation errors for the 4 protocols fit with gkm or gkm+disp using D+M‐ signals (kernel sharpness s=s0) over a prolonged ATT range. (A): CBF errors fit with gkm; (B): CBF errors fit with gkm+disp; (C): ATT errors fit with gkm; (D): ATT errors fit with gkm+disp FIGURE S4 Arterial blood volume (aBV) estimation means and standard deviations for the 3 multi‐PLD protocols fitted with gkm+mvc using D‐M+ signals. The dashed magenta line indicates identity FIGURE S5 Simulation CBF and ATT estimation error means and standard deviations for the 4 protocols fitted with gkm or gkm+mvc using D‐M‐ signals over a prolonged ATT range FIGURE S6 Simulation CBF and ATT estimation error standard deviations for the 4 protocols fitted with gkm over a prolonged ATT range. (A): CBF error std using D‐M‐; (B): CBF error std using D‐M+; (C): ATT error std using D‐M‐; (D): ATT error std using D‐M+. The dashed magenta line indicates the upper limit of the range of ATT that CBF‐ATTopt and CBFopt was optimised for FIGURE S7 Simulation CBF and ATT estimation errors for the 3 normal‐range protocols and 3 prolonged‐range protocols fit with gkm over a range of aBV using D‐M+ signals. ATT was held constant at 1.4s in simulation across all aBVs. (A): CBF errors of normal‐range protocols; (B): [file MRM-86-2208-s001.docx]

**Supplementary Materials**


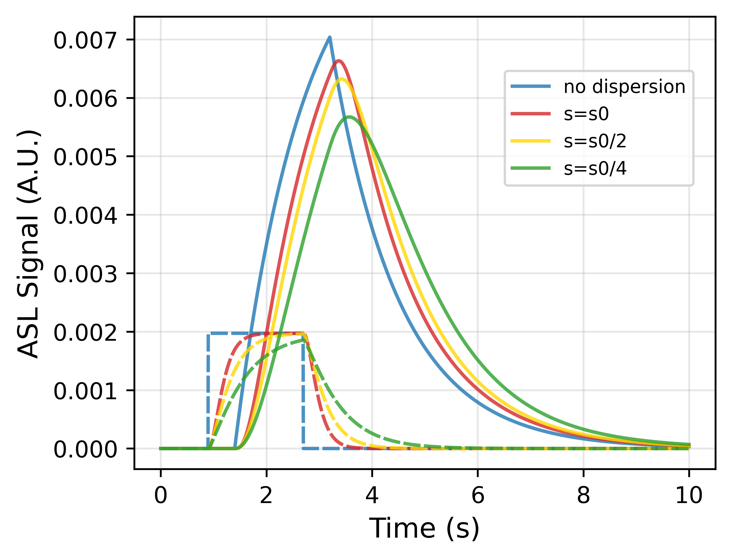


Figure S1. The effect of different dispersion kernels to the tissue (solid lines) and macrovascular signals (dashed lines). A smaller parameter $s$ indicates a higher level of flow dispersion. Parameters used in this simulation: tissue ATT = 1.4s, macrovascular ATT = 0.9s.


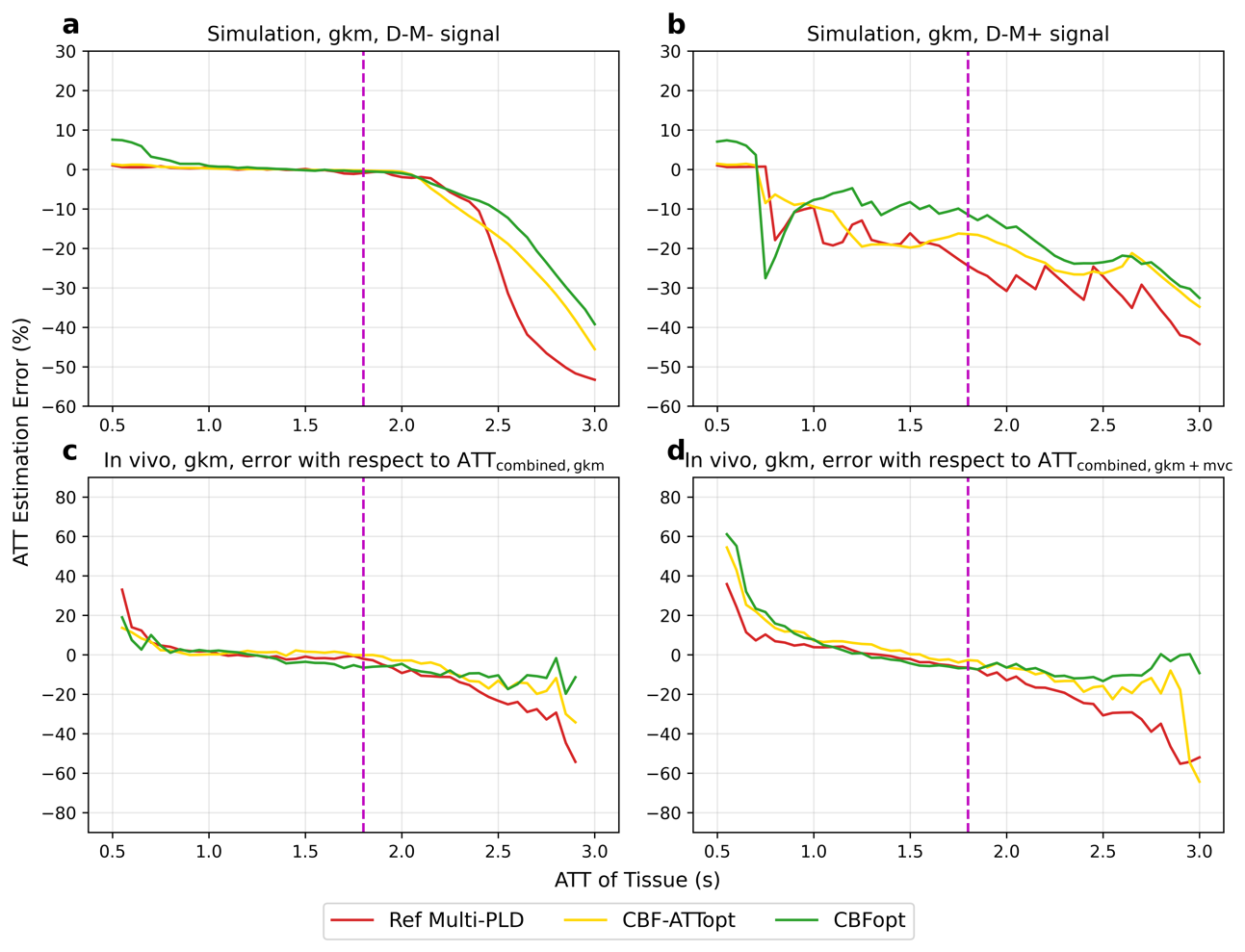


Figure S2. ATT estimation errors for the 3 multi-PLD protocols fitted with gkm over a prolonged ATT range. (a): simulation data using D-M-; (b): simulation data using D-M+; (c): *in vivo* estimation error with respect to ATT_combined, gkm_; (d): *in vivo* estimation error with respect to ATT_combined, gkm+mvc_. The dashed magenta line indicates the upper limit of the range of ATT that CBF-ATTopt and CBFopt was optimised for.


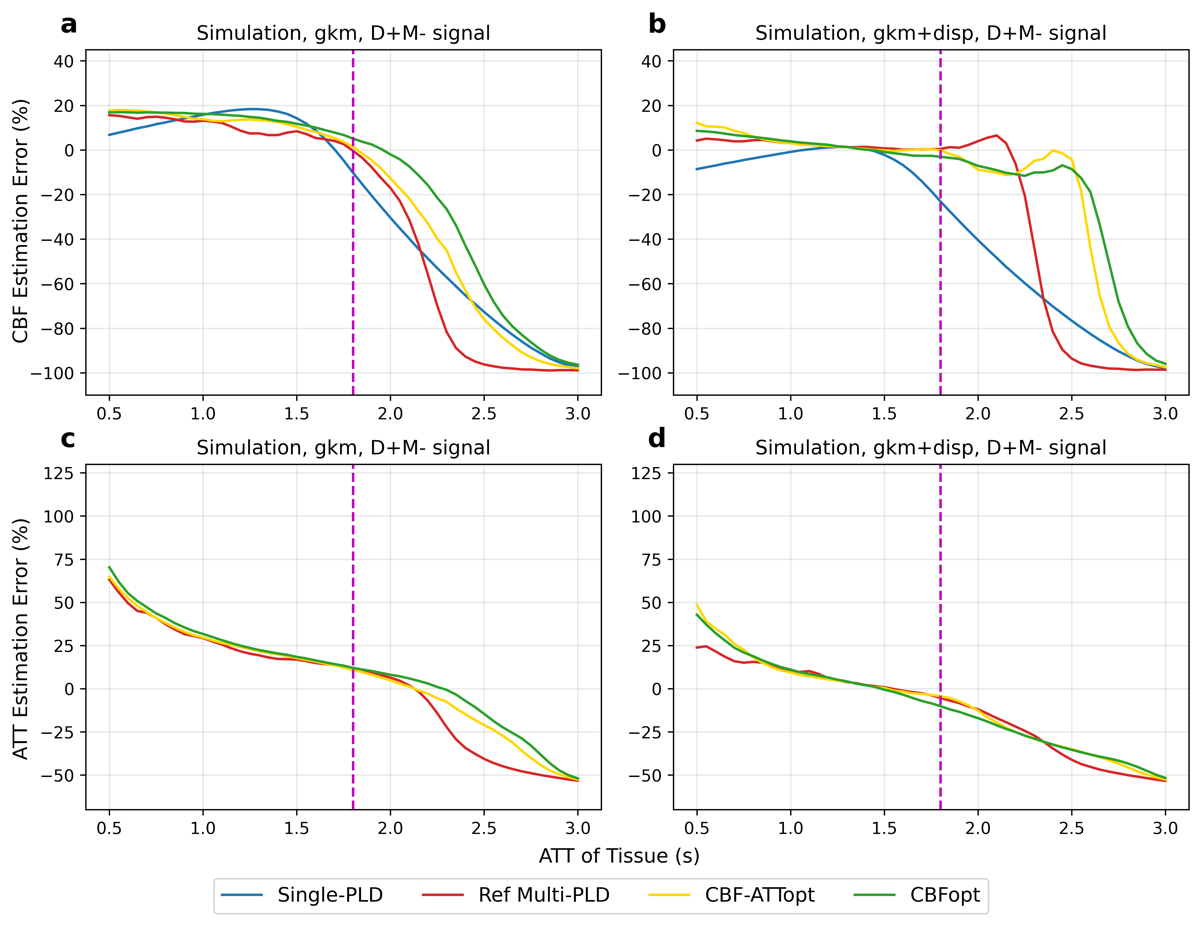


Figure S3. Simulation CBF and ATT estimation errors for the 4 protocols fit with gkm or gkm+disp using D+M- signals (kernel sharpness $s=s_{0}$) over a prolonged ATT range. (a): CBF errors fit with gkm; (b): CBF errors fit with gkm+disp; (c): ATT errors fit with gkm; (d): ATT errors fit with gkm+disp.


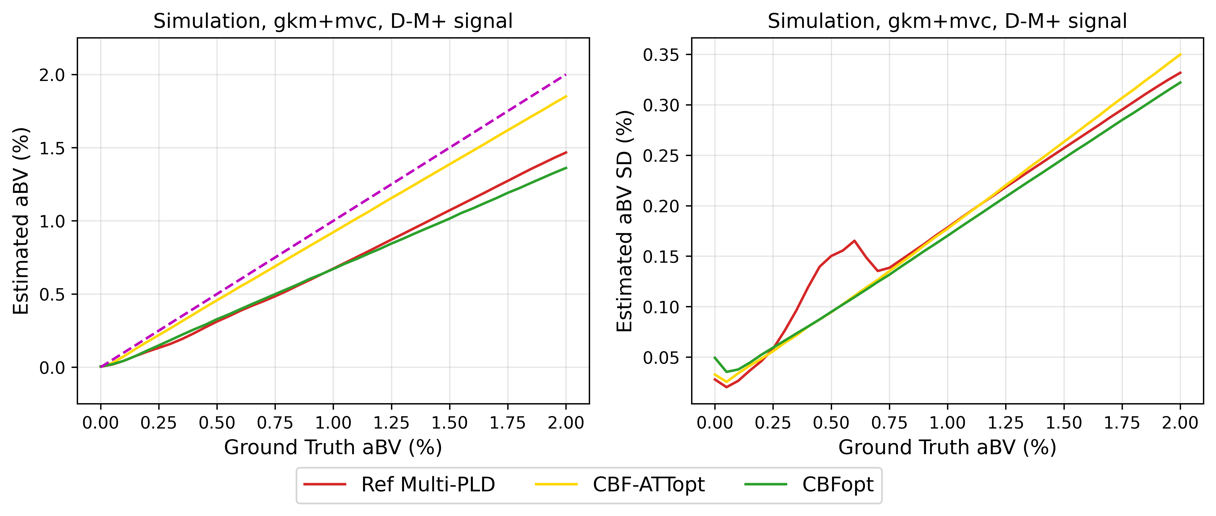


Figure S4. Arterial blood volume (aBV) estimation mean and standard deviation for the 3 multi-PLD protocols fitted with gkm+mvc using D-M+ signals. The dashed magenta line indicates identity.


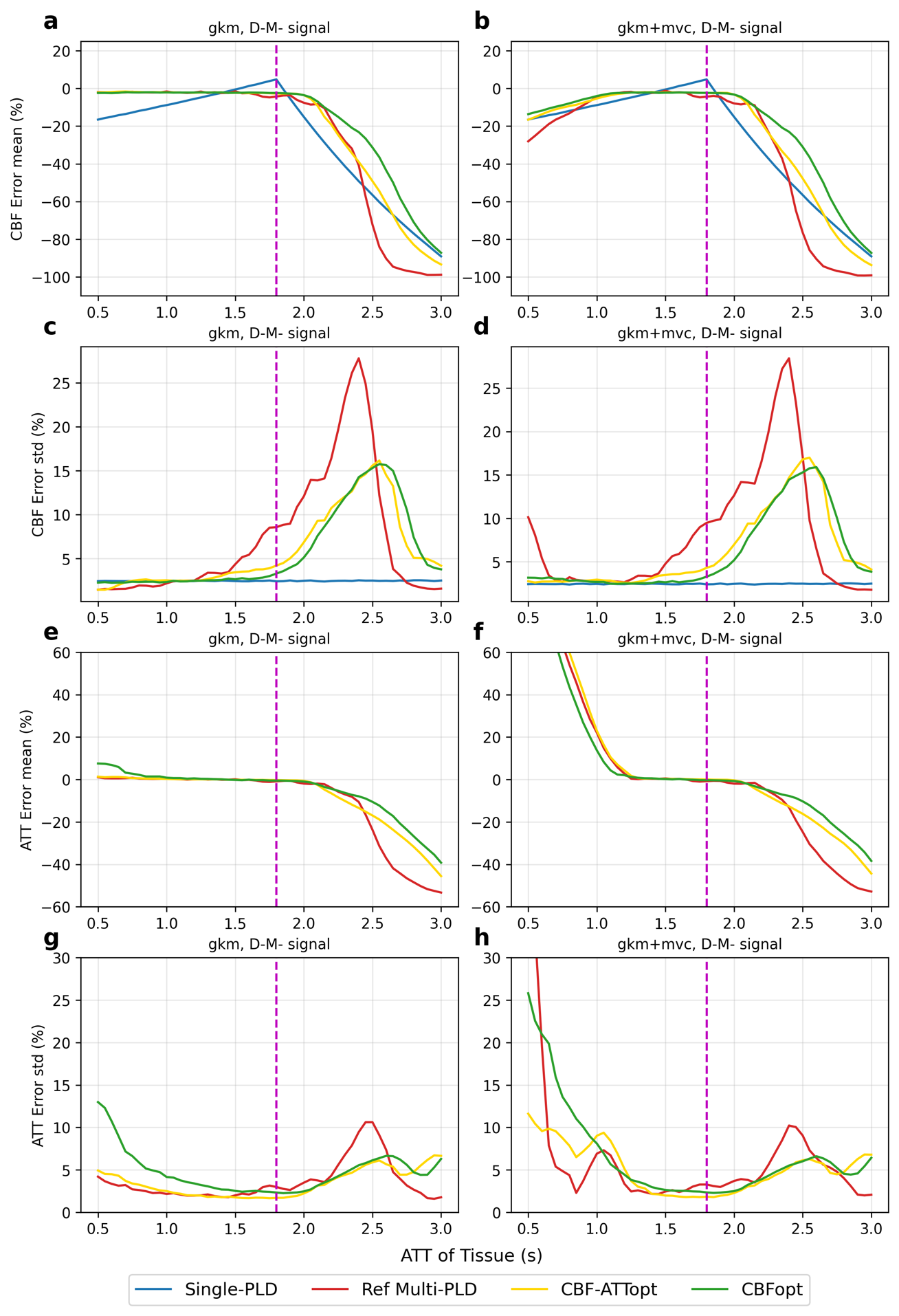


Figure S5. Simulation CBF and ATT estimation error means and standard deviations for the 4 protocols fitted with gkm or gkm+mvc using D-M- signals over a prolonged ATT range.


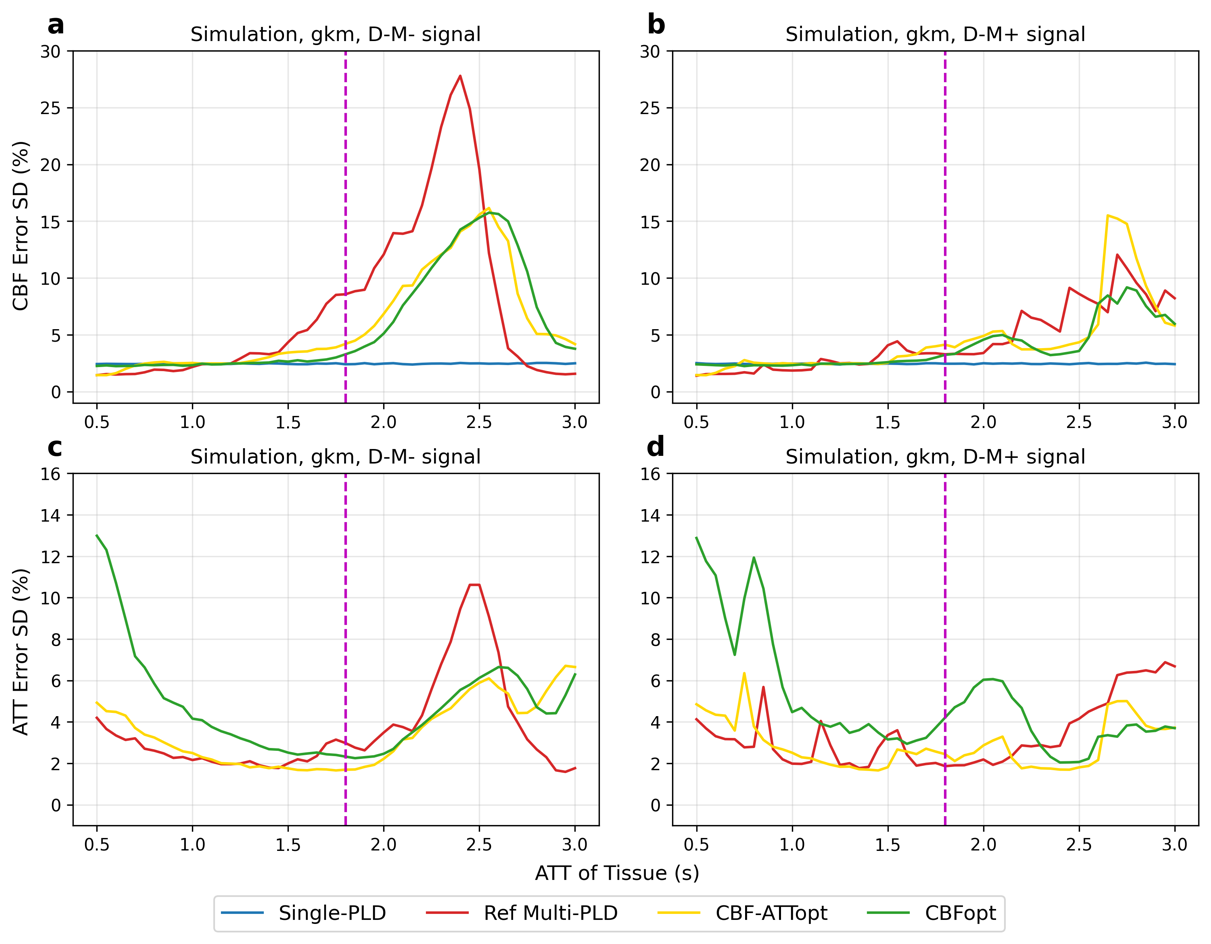


Figure S6. Simulation CBF and ATT estimation error standard deviations for the 4 protocols fitted with gkm over a prolonged ATT range. (a): CBF error std using D-M-; (b): CBF error std using D-M+; (c): ATT error std using D-M-; (d): ATT error std using D-M+. The dashed magenta line indicates the upper limit of the range of ATT that CBF-ATTopt and CBFopt was optimised for.


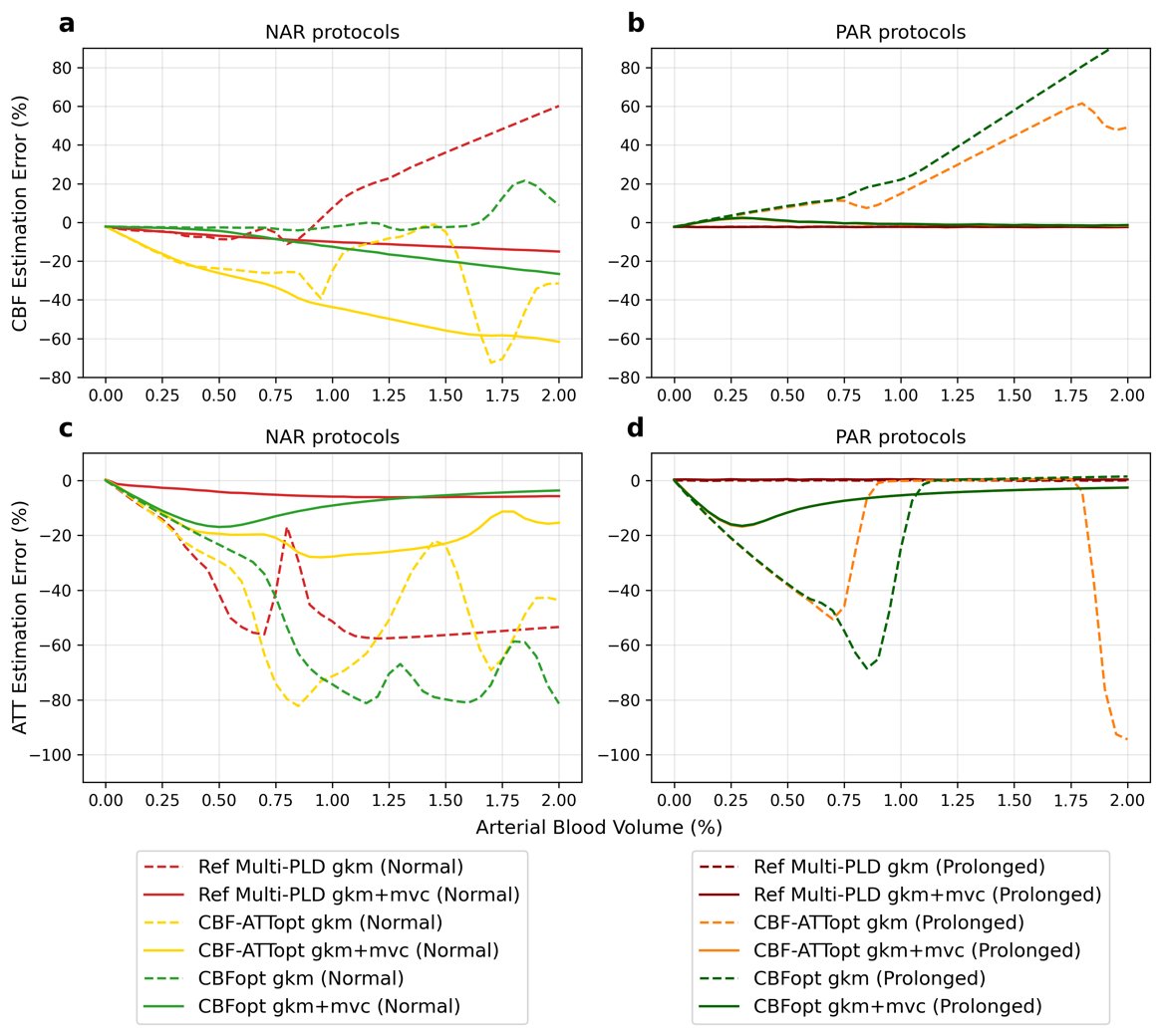


Figure S7. Simulation CBF and ATT estimation errors for the 3 normal-range protocols and 3 prolonged-range protocols fit with gkm over a range of aBV using D-M+ signals. ATT was held constant at 1.4s in simulation across all aBVs. (a): CBF errors of normal-range protocols; (b): CBF errors of prolonged-range protocols; (c) ATT errors of normal-range protocols; (d): ATT errors of prolonged-range protocols.


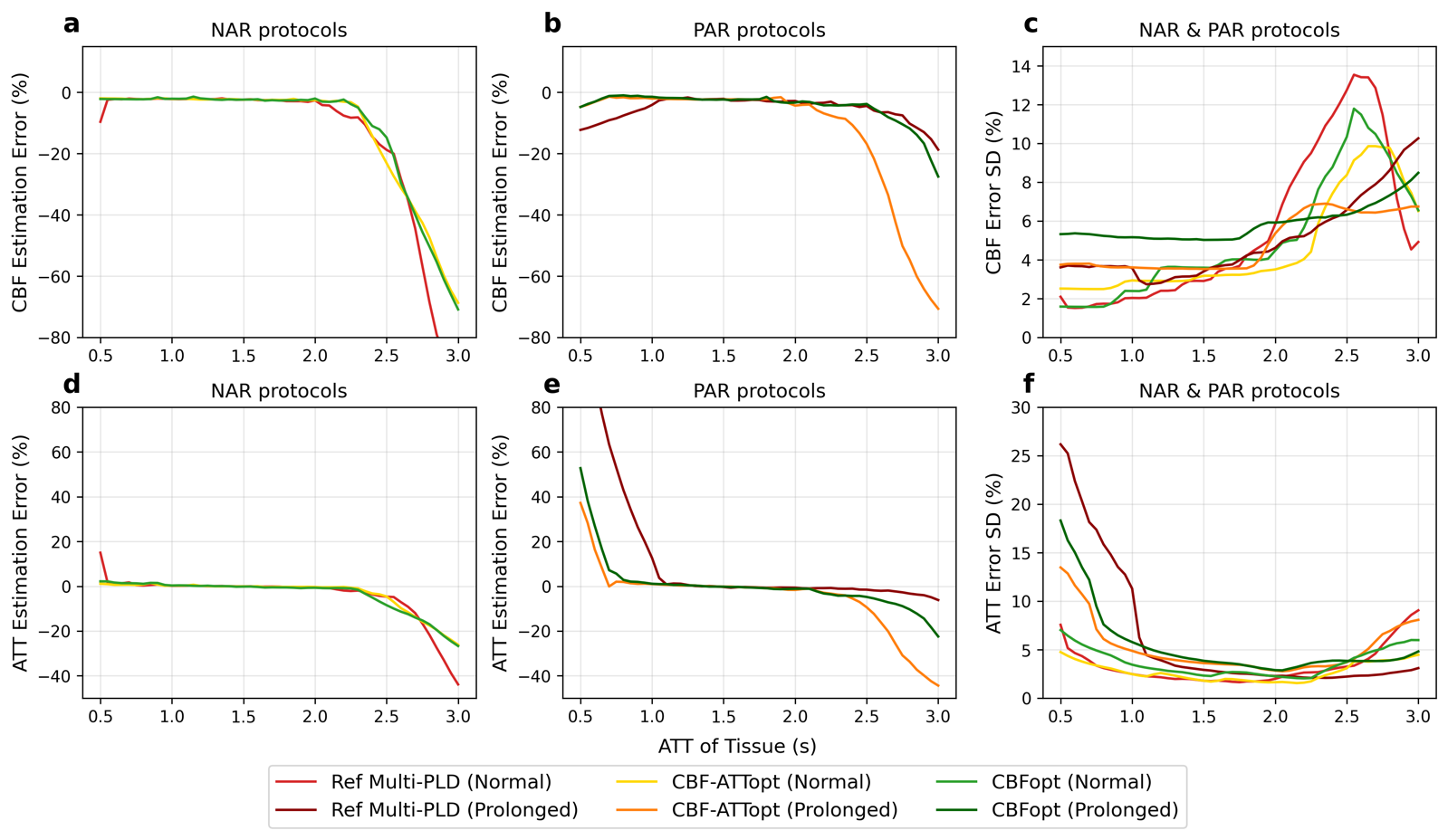


Figure S8. Simulation CBF and ATT estimation error means and standard deviations for the 3 normal-ATT-range (NAR) protocols and 3 prolonged-ATT-range (PAR) protocols fitted with gkm over a prolonged ATT range using D-M- signals. (a): CBF errors of NAR protocols; (b): CBF errors of PAR protocols; (c) CBF errors std of NAR & PAR protocols; (d) ATT errors of NAR protocols; (e): ATT errors of PAR protocols; (f): ATT errors std of NAR & PAR protocols.

| **Parameter** | **Mean** | **Std** |
| --- | --- | --- |
| $f (ml/100g/min)$ | 0 | 10^3^ |
| $\Delta t \left( s \right)$ | 1.3 | 10^3^ |
| $\tau\left( s \right)$ | 1.8 | 0.3 |
| $aBV \left( \% \right)$ | 0 | 10^3^ |
| $\Delta t_{a} \left( s \right)$ | 0.9 | 10^3^ |
| $s_{0} \left( s^{-1} \right)$ | 1/0.13 | 0.3 |
| $p_{0} \left( s \right)$ | 0.17 | 0.3 |
| $T_{1b} \left( s \right)$ | 1.65 | 0.3 |
| $T_{1t} (s)$ | 1.3 | 0.3 |

Table S1. Specifications of the estimation priors (as mean and standard deviation by a normal distribution) used in the variational Bayesian inference method for simulation experiments.

| **Protocol** | **Post-labelling delays (s)** | **PLDs (N)** | **Averages (N)** |
| --- | --- | --- | --- |
| $0.5\leq ATT\leq2.0s$ | | | |
| Reference multi-PLD | 0.5, 0.725, 0.925, 1.15, 1.35, 1.575, 1.775, 2 | 8 | 6 |
| CBF-ATTopt | 0.2, 0.9, 0.9, 1.15, 2, 2.2 | 6 | 8 |
| CBFopt | 0.2, 0.925, 1.425, 1.85, 2.025, 2.15, 2.25, 2.3, 2.3, 2.3 | 10 | 4 |
| $1.0\leq ATT\leq3.0s$ | | | |
| Reference multi-PLD | 1, 1.25, 1.5, 1.75, 2, 2.25, 2.75, 3 | 9 | 4 |
| CBF-ATTopt | 0.7, 1.875, 1.9, 1.9, 1.925, 2.75, 2.95, 3.1, 3.3 | 9 | 4 |
| CBFopt | 0.7, 1.8, 2.5, 2.875, 3.075, 3.3, 3.3, 3.325 | 8 | 4 |

Table S2. Protocol timings of 3D readout from Woods *et al^7^*. Two sets of protocols were developed, one for a normal range of $0.5\leq ATT\leq2.0s$, the other one for a prolonged range of $1.0\leq ATT\leq3.0s$.
